# Mixed Adjuvant Formulations Reveal a New Combination That Elicit Antibody Response Comparable to Freund's Adjuvants

**DOI:** 10.1371/journal.pone.0035083

**Published:** 2012-04-11

**Authors:** Rachel P. J. Lai, Michael S. Seaman, Paul Tonks, Frank Wegmann, David J. Seilly, Simon D. W. Frost, Celia C. LaBranche, David C. Montefiori, Antu K. Dey, Indresh K. Srivastava, Quentin Sattentau, Susan W. Barnett, Jonathan L. Heeney

**Affiliations:** 1 Department of Veterinary Medicine, University of Cambridge, Cambridge, United Kingdom; 2 Division of Viral Pathogenesis, Beth Israel Deaconess Medical Center, Boston, Massachusetts, United States of America; 3 The Sir William Dunn School of Pathology, University of Oxford, Oxford, United Kingdom; 4 Department of Surgery, Duke University Medical Center, Durham, North Carolina, United States of America; 5 Novartis Vaccines and Diagnostics Inc., Massachusetts, United States of America; University of Rochester, United States of America

## Abstract

Adjuvant formulations capable of inducing high titer and high affinity antibody responses would provide a major advance in the development of vaccines to viral infections such as HIV-1. Although oil-in-water emulsions, such as Freund's adjuvant (FCA/FIA), are known to be potent, their toxicity and reactogenicity make them unacceptable for human use. Here, we explored different adjuvants and compared their ability to elicit antibody responses to FCA/FIA. Recombinant soluble trimeric HIV-1 gp140 antigen was formulated in different adjuvants, including FCA/FIA, Carbopol-971P, Carbopol-974P and the licensed adjuvant MF59, or combinations of MF59 and Carbopol. The antigen-adjuvant formulation was administered in a prime-boost regimen into rabbits, and elicitation of antigen binding and neutralizing antibodies (nAbs) was evaluated. When used individually, only FCA/FIA elicited significantly higher titer of nAbs than the control group (gp140 in PBS (p<0.05)). Sequential prime-boost immunizations with different adjuvants did not offer improvements over the use of FCA/FIA or MF59. Remarkably however, the concurrent use of the combination of Carbopol-971P and MF59 induced potent adjuvant activity with significantly higher titer nAbs than FCA/FIA (p<0.05). This combination was not associated with any obvious local or systemic adverse effects. Antibody competition indicated that the majority of the neutralizing activities were directed to the CD4 binding site (CD4bs). Increased antibody titers to the gp41 membrane proximal external region (MPER) and gp120 V3 were detected when the more potent adjuvants were used. These data reveal that the combination of Carbopol-971P and MF59 is unusually potent for eliciting nAbs to a variety of HIV-1 nAb epitopes.

## Introduction

One of the major challenges in developing vaccines against highly variable viruses, such as HIV-1 and HCV, is the relatively poor immunogenicity of the subunit Envelope glycoprotein (Env) antigens, which fail to elicit antibody responses that protect against the majority of circulating viral strains. Early studies on HIV-1 indicated an inverse correlation between antibody binding avidity and the frequency of transmission [1], [2], [3]. In order to generate high avidity antibodies, antigen-activated B cells need to undergo extensive somatic hypermutation. It has been demonstrated during the immune response to HIV-1 infection, that somatic hypermutation is essential to increase antibody affinity and neutralization breadth and potency [4]. Indeed, analyses on some of the broadly neutralizing antibodies (bnAbs) against HIV-1 reveal that these bnAbs undergo 19–46% more somatic hypermutation than their weakly or non-neutralizing counterparts [5], [6], [7], [8], [9], [10]. Given these observations, an ideal vaccine should, therefore, include an adjuvant that can drive somatic hypermutation, in the hope of eliciting high avidity antibodies with increased breadth and potency.

Adjvuants are substances capable of stimulating an immune response to an antigen. Due to their immunopotentiating and immunostimulatory properties, adjuvants can greatly reduce the dose and number of immunizations required to achieve sustained protective immunity [11], [12], [13], [14], [15], [16], which is of particular importance during pandemic outbreaks. A central issue in developing an adjuvant suitable for clinical use is possible risk of local and systemic reactogenicity and toxicity. For example, Freund's complete adjuvant (FCA), which is a paraffin oil-in-water emulsion containing heat-inactivated bacterial products (*M. tuberculosis* or *M. butryicum*), is known to be very potent and is often used for generation of high titer antisera in animals [17]. However FCA is toxic and causes cell and tissue damage upon injection. To reduce toxicity, bacterial components were removed in related oil-in-water adjuvants, such as Freund's incomplete adjuvant (FIA) and Montanide ISA [18], [19]. Immunization of malarial or HIV-1 antigens in Montanide ISA induced both nAbs and CD8^+^ T-cell responses [20], [21], [22], [23]. However, a number of vaccinees in these studies developed prolonged pain and sterile abscess formation at the injection site or experienced severe systemic reactions, suggesting that the adjuvant is not suitable for use in human vaccines [20], [21], [22], [23].

Because of their respectable safety record, aluminum-based salts (alums) remain the dominant adjuvants allowed for human use since their initial successful formulation for tetanus toxin vaccine [24]. However, alum is only weakly stimulatory especially when compared to FCA/FIA [25], and since part of its function is to precipitate antigen, this may adversely affect the antigenicity of conformationally-sensitive antigens. Hence, substantial efforts have been devoted in recent years to developing a new generation of potent and safe adjuvants. It was not until 1997 that MF59 was licensed for use in the seasonal influenza vaccine for the elderly in Europe, the first new adjuvant licensed since alum [26]. MF59 is a squalene oil-in-water emulsion stabilized by Tween-80 and Span 85 surfactants [26], [27]. It was found to cause significant local immune stimulation and recruit and increase antigen uptake by dendritic cells (DCs) [28], [29], [30]. Early studies in baboons and humans showed that MF59 offered superior potency over alum with an HBV vaccine [31], [32]. Subsequent clinical trials with CMV, HSV and HIV-1 candidate vaccines indicated that MF59 was well tolerated in human volunteers including infants, and enhanced humoral as well as T-helper responses [33], [34], [35], [36], [37].

In addition to oil-in-water emulsions, many other types of experimental adjuvant are under intensive evaluation, including synthetic polymers. Polyanionic carbomers, such as Carbopols, have long been used in pharmaceuticals as controlled release agents for oral tablets or as bioadhesives for topical or trans-mucosal drug delivery [38], [39]. Pilot studies of experimental veterinary vaccines with different types of Carbopol adjuvant have shown increased antibody responses with low reactogenicity [40], [41], and one of the carbomers, Carbopol-941P, is licensed for vaccine use in pigs (Suvaxyn, Pfizer) [42], [43]. The adjuvanticity of two other polyanionic carbomers, Carbopol-971P and Carbopol-974P, was recently analyzed by others [44], [45]. Both polymers are networks of primary polyacrylic acid chains interconnected by crosslinks. The 971P formulation has lower viscosity and fewer crosslinking sites than the 974P formulation [46], [47].

Here, we compare a number of adjuvants, including FCA/FIA, MF59, Carbopol-971P and 974P in the context of soluble recombinant HIV-1 gp140 antigen, by systemic immunization of rabbits. Based on our findings, we propose that specific adjuvant combinations drive antibody responses more effectively than using adjuvants singly.

## Materials and Methods

### Ethics statement

The animal study was carried out in strict accordance with the UK Animals (Scientific Procedure) Act 1986, and the protocol was approved by the local Ethical and Welfare Committee of the University of Cambridge and the UK Home Office (Project license no. 80/2238).

### Recombinant gp140 purification

HIV-1 gp140_SF162_ has been previously characterized and was obtained as culture supernatant from Novartis Vaccines & Diagnostics, USA. The gp140 glycoprotein was purified using a published protocol [48]. Briefly, supernatant was loaded onto a *Galanthus nivalis* agarose affinity column equilibrated with buffer (20 mM Tris, 100 mM NaCl, pH 7.4), and bound gp140 was eluted with 500 mM methyl mannose pyranoside in equilibration buffer. The eluate was then loaded onto a DEAE column equilibrated with buffer (20 mM Tris, 100 mM NaCl, pH 8.0), such that contaminants were bound to the column while the gp140 flowed through. The flow-through was adjusted to pH 6.8 with 10 mM NaPO_4_ before loading onto a ceramic hydroxyapatite (CHAP) column that has been previously equilibrated with buffer (10 mM Na_2_HPO_4_, 100 mM NaCl, pH 6.8). The flow through, which contained the purified glycoprotein, was collected and then concentrated to 1 µg/µl using an AMICON YM-30 (30 kDa cut-off) ultrafiltration disc (Millipore, UK) and stored at −80°C until use.

### Adjuvant-immunogen formulation

The gp140_SF162_ (25 µg per animal) was formulated with different adjuvant(s) immediately prior to immunization. A detailed example on the dosages for each adjuvants and antigen is shown in Table S1. In order to keep the final volume identical in all groups, 25 µg of gp140_SF162_ (1 µg/µl) was first diluted 1∶1 (v/v) with PBS, or 25 µl glycoprotein to 25 µl of PBS, before mixing 1∶1 (v/v) with Freund's complete or incomplete adjuvant (Sigma-Aldrich, UK), or MF59 (Novartis Vaccines & Diagnostics, USA). The mixture, with a final volume of 100 µl (per animal), was then incubated for 10 min before injection. For Carbopol-971P (Lubrizol, USA) and 974P (Particle Sciences, USA), both polymers were first prepared to a 2% (w/v) suspension with enough PBS, before mixing 1∶1 (v/v), or 25 µl to 25 µl, with gp140_SF162_. The mixture was then brought to the same final volume of 100 µl with PBS and incubated for 30 min before injection [44], [45]. For the combination groups, gp140 was first mixed 1∶1 (v/v), or 25 µl to 25 µl, with 2% Carbopol-971P/974P and incubated for 30 min. The mixture was then added 1∶1 (v/v) with 50 µl of MF59 and incubated for another 10 min before injection. For the no adjuvant group, 75 µl of PBS was added to gp140_SF162_ (25 µl) to make the final volume 100 µl for the injection. Due to the high viscosity of the adjuvants, substantial vortexing was required during the formulation process to ensure the antigen-adjuvant mixture was homogenized before use.

### Protein gel electrophoresis

The conformation of the gp140, in the presence or absence of adjuvants, was assessed by reducing, non-reducing and Blue-Native (BN) polyacrylamide gel electrophoresis using standard protocols. Briefly, gp140_SF162_ was formulated with or without adjuvant(s) as described above. To separate the glycoprotein from the adjuvant(s), the mixture was centrifuged at high speed for 30 min and the aqueous phase, which contained the glycoprotein, was extracted and quantified with a spectrophotometer. The extracted glycoprotein was then denatured at 90°C and reduced with beta-mercaptoethanol (βME) for reducing SDS-PAGE. The glycoproteins were not heat denatured or treated with βME for the non-reducing condition. Coomassie Blue dye was added to 5 µg of gp140 and then separated in the absence of sodium dodecyl sulphate for BN-PAGE.

### Antibody binding assay

The effect of adjuvant on antigenicity was also analyzed by antibody binding. Briefly, gp140_SF162_ was first formulated with adjuvant(s) and then extracted before being coated (0.5 µg/well) to microwell plates for 2 h at 16°C. The plates were washed 3 times with 1× PBS and blocked with 5% BSA for 30 min, followed by another 3 washes with PBS. Monoclonal antibodies labeled with Europium (Perkin Elmer, UK) were added (100 ng/well) and incubated for 1 h at 16°C. The plates were washed 3 times with wash buffer (25 mM Tris-HCl pH 7.8 containing 1% Triton X-100). Enhancement solution (200 µl/well; Perkin Elmer) was added and the plates were developed for 15 min and the Relative Luminescence Unit (RLU) emitted were measured with a 1420 Victor Multilabel Counter (Perkin Elmer, UK).

### Immunization and sera collection

Twelve groups of 6 New Zealand white female rabbits each received 3 intramuscular immunizations of gp140_SF162_ at week 0, 4 and 12 (Figure S1). Except for animals in group 1, in which 75 µg/injection of gp140 was used, all other animals received 25 µg of gp140 for each immunization. Animals in both groups 1 and 2 received FCA for the first immunization and FIA for the next two. Animals in groups 3–5 received MF59, Carbopol-971 or, Carbopol-974P for all three injections, respectively. For groups 6 and 7, rabbits received either Carbopol-971P or Carbopol-974P for the first injection, followed by MF59 for the next two. The regimen was reversed for groups 8 and 9, in which animals were injected twice with MF59, followed by a final injection of either Carbopol-971P or Carbopol-974P. For group 10, animals received a mixture of Carbopol-971P and MF59 adjuvants for all three immunizations. Similarly, animals in group 11 received a mixture of Carbopol-974P and MF59 for all three immunizations. Finally, animals in group 12 received only gp140_SF162_ for all three injections, without any adjuvant. Serum samples were collected 2 weeks before and after each immunization at week −2, 2, 6 and 14, and a terminal sample was collected at week 16. One of the animals in group 1 died before the end of the study of cause unrelated to adjuvant toxicity.

### Endpoint titer and avidity index ELISA

The endpoint titers of Env-specific antibodies in the sera were measured by standard ELISA. Briefly, 96 maxisorp microwell plates (Nunc, UK) were coated overnight with purified gp140_SF162_ (50 ng/well) in 50 mM sodium carbonate-bicarbonate buffer (pH 9.6) at 4°C, and then blocked with PBS containing 0.05% Tween-20 and 5% non-fat milk for 2 h at 16°C. Serum samples were serially diluted (2-fold) and 50 µl were added to triplicate wells and incubated for 1 h at 16°C, and then washed 3 times with wash buffer (PBS containing 0.05% Tween-20). A rabbit IgG specific secondary antibody conjugated to HRP (Sigma-Aldrich, UK), diluted 1∶10,000, was added and incubated for 1 h at 16°C. After three washes with wash buffer, the plate was developed with a 1-Step 3,3,5,5-tetramethylbenzidine (Pierce Thermo Scientific, UK) for 20 min in the dark before being stopped with 2 M H_2_SO_4_. Absorbance values were detected at 450 nm using a Bio-Rad iMark Microplate Reader (Bio-Rad, UK).

The avidity indices of vaccinated sera were measured by its resistance to 8 M urea in binding to gp140_SF162_ antigen [49]. Briefly, microwell plates were prepared and blocked as described above. Serum samples were diluted to give an OD_450 nm_ readout between 1.0 and 1.5 in endpoint ELISA and were added to two sets of triplicate wells to incubate for 1 h at 16°C. The wells were then washed three times with either PBS-Tween or 8 M urea in PBS-Tween, before incubating with an anti-rabbit secondary antibody. The plates were washed and developed with TMB as described above. The avidity index was calculated as the percentage of average urea treated OD_450 nm_/average PBS-Tween OD_450 nm_. Antisera with index values >50% were designated high avidity, 30–50% were designated intermediate avidity and <30% were designated low avidity.

### Real-time neutralizing epitope competition assay

*Galanthus nivalis* lectin (GNA) was coated at 100 ng/well onto microwell plates in PBS buffer at 4°C overnight. The microwell plates were blocked with 2% BSA for 2 h, before gp140_SF162_ (100 ng/well) was added in triplicate and incubated for 2 h at 16°C. The microplate was washed 3 times with wash buffer (25 mM Tris-HCl pH 7.8 containing 1% Triton X-100). To determine the approximate epitopes that the polyclonal sera were raised to, antisera from vaccinated animals were diluted according to their endpoint titer to approximately 1×10^5^ IgG to compete with Eu^3+^-labeled mAbs (100 ng/well) for binding to antigens. Unbound antisera and mAbs were removed by 6 washes. Enhancement solution (Perkin Elmer, UK) was added and incubated for 20 min and the RLU_620 nm_ was measured in a Envision luminometer (Perkin Elmer, UK). Avidity of the antisera to the epitopes was determined by reduction in the Eu^3+^ luminescence intensity of the specific mAb that the serum was competing with. The 2F5 and 4E10 mAbs were purchased from Polymun (Vienna, Austria) and other mAbs were kindly provided upon request (see Acknowledgements).

### Neutralization assay

The TZM-bl assay was carried out using a standard protocol with molecularly cloned pseudoviruses [50]. Briefly, pseudovirus was incubated with serially diluted rabbit antisera for 1 h at 37°C before being placed into wells of 96-well plates seeded with TZM-bl cells. After 48 h incubation at 37°C, the cells were lysed and luciferase signal in the lysate was developed with Britelite Plus substrate (1∶1 v/v; Perkin Elmer) and read in a luminometer.

### Statistical analysis

Comparison of log transformed binding and avidity data between groups was tested using a one way ANOVA, with a correction for multiple comparisons between groups conducted using Tukey's Honestly Significant Differences. Comparison of log-transformed neutralization titers between groups was performed using a linear mixed effects model, fitted using a Bayesian Markov Chain Monte Carlo approach. Markov chains were run for 110,000 iterations, with a burnin of 10,000 steps, and the resulting chain was thinned to provide 1,000 samples of parameter values.

## Results

### Conformation and antigenicity of gp140 in adjuvant formulations

To examine if the adjuvants modified antigen conformation, gp140_SF162_ was formulated in a single adjuvant or a combination of adjuvants described in the immunization protocol in Materials and Methods. The samples were extracted and analyzed for conformational changes and antigenicity by gel electrophoresis and antibody binding ELISA.

**Figure 1:**
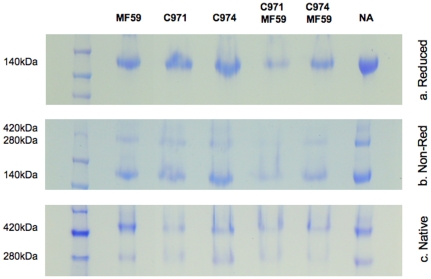
gp140_SF162_ conformation upon formulation with adjuvant. The gp140_SF162_ glycoprotein was formulated with or without adjuvants and assayed on PAGE gel for conformation. (a) Regardless of presence or absence of adjuvants, a single band at 140 kDa was observed in the reduced gel. (b) Similar observation was made in the non-reduced gel, where all the samples showed two bands at 140 kDa and 280 kDa, representing the non-crosslinked and disulfide crosslinked gp140 trimers, respectively. (c) A BN-PAGE gel was used to examine the native conformation of gp140 in the presence of adjuvants. None of the adjuvants appear to affect the structure of gp140.

When gp140_SF162_ was formulated in FCA or FIA, there was unavoidable and substantial spillage of the samples from the wells during loading, particularly for the former. To extract only the aqueous gp140 from the mineral oil, samples were centrifuged at high speed and the oil phase was removed. However, a smearing separation was still observed in both reduced and non-reduced, as well as BN-PAGE gels for these two samples, making interpretation difficult (data not shown). We observed no biophysical differences with any of the other adjuvants, including MF59, in either reducing or non-reducing gels, compared to the unadjuvanted gp140 (Figures 1a & 1b). Similarly, no differences were observed when samples were assayed by BN-PAGE (Figure 1c).

**Figure 2:**
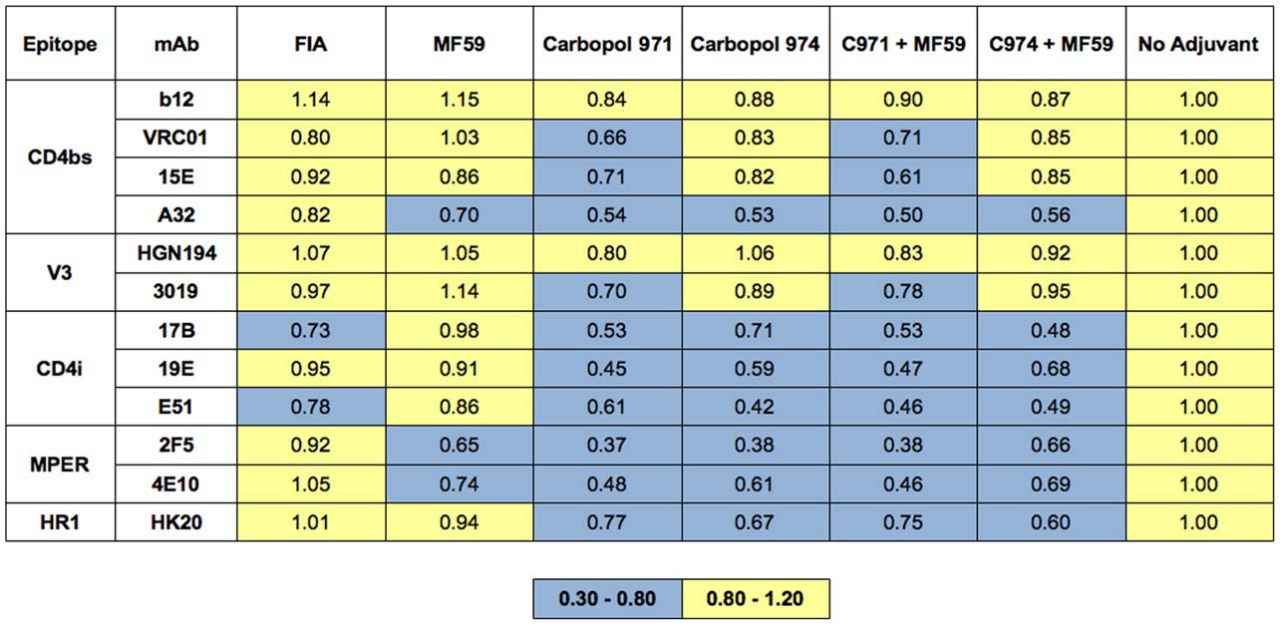
Monoclonal antibody binding to gp140_SF162_ in the presence of adjuvant. The antigenicity of gp140_SF162_ in the presence of adjuvants was measured by mAb binding. A panel of 12 different mAbs, representing different epitope domains of the Env, was used. The signal of mAb binding to the adjuvant-formulated gp140_SF162_ was calculated relative to the signal measured in the unadjuvanted sample, which is assigned to have the value of 1. The data is color coded: Yellow represents no difference in mAb binding, or no change of antigenicity as compared to the unadjuvanted sample and light blue represents up to 3-fold decrease of mAb binding (or antigenicity). Both FIA and MF59 adjuvants appeared to have little effect on antigenicity, as mAb binding was very similar to the unadjuvanted sample. On the other hand, decreased mAb bindings were detected in the presence of both of the Carbopols.

To further analyze whether gp140_SF162_ underwent conformation changes that could affect its antigenicity in the presence of adjuvants, we assayed for binding of a panel of mAbs to the antigen by ELISA. The aqueous glycoprotein was separated from the oil phase from samples containing FCA, FIA or MF59. The amount of gp140_SF162_ extracted was quantified and normalized before coating for ELISA. There was significant loss in mAb binding in the presence of FCA (data not shown). We suspected this was due to the high viscosity and hydrophobicity of the residual mineral oil, which either compromised gp140 binding to the plate, or heavily masked most of the epitopes and prevent mAb from accessing them. On the contrary, in the presence of FIA, which is substantially less viscous, we observed no change in antigenicity of the extracted gp140 as compared to the unadjuvanted sample (Figure 2). MF59, the other oil-in-water emulsion that also has lower viscosity than FCA, showed no significant changes in gp140 antigenicity as well.

In contrast to the oil-in-water emulsions, gp140_SF162_ could not be separated from the Carbopol polymers since both the antigen and the adjuvants were in aqueous phase. Formulation with Carbopol-971P alone, or in a mixture with MF59, did not appear to interfere binding of b12 (CD4bs) or HGN194 (V3 loop), while modest reduction was observed with other mAbs targeting the same or other antigenic sites (Figure 2). Similar result was also demonstrated in a recent study, where mAbs b12 and CD4IgG2 showed indistinguishable level of binding to both native and Carbopol-971P formulated gp140, while binding of 17b (CD4i) and F425-B4e8 (V3 loop) was significantly compromised [45]. Since Carbopol is polyanionic, its interaction with the positively charged side chains of gp140 might induce partial masking and prohibit mAb binding to its epitope. Nevertheless, further investigation will be required to study the molecular interaction between the carbomers and the antigen for an accurate interpretation.

### Combination of Carbopol-971P and MF59 elicited binding titers comparable to Freund's adjuvants

Twelve groups of 6 rabbits each were immunized 3 times with gp140_SF162_ with different adjuvant regimens (Figure S1). Minor and transient swelling with reddening at the injection site were observed in rabbits that received FCA/FIA, which was resolved within days. No local or systemic reactogenicity was observed in rabbits that received other adjuvant formulations.

**Figure 3:**
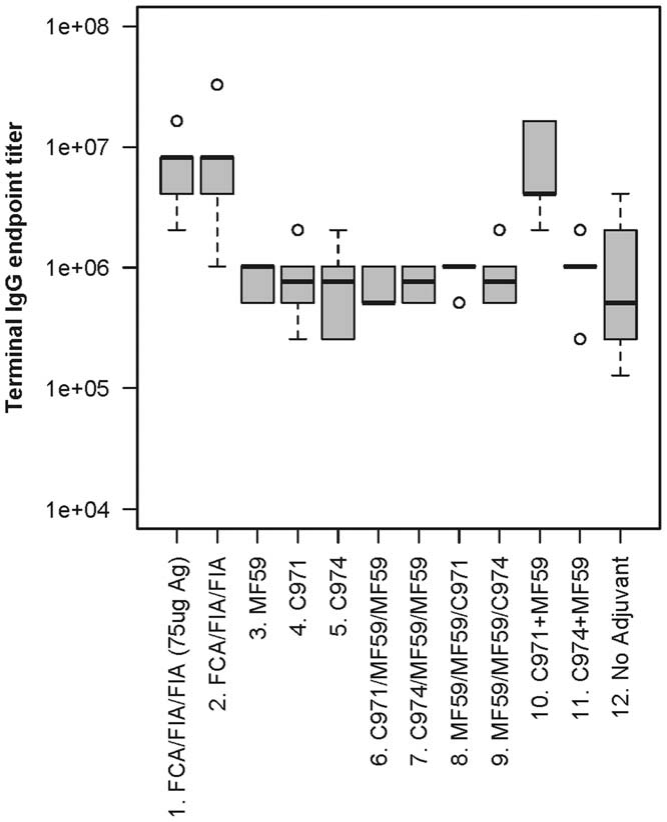
Endpoint IgG binding titer. The antigen-specific IgG titer was measured by ELISA. Antiserum (terminal time point) was serially diluted (2-fold) and the endpoint titer was defined as the last dilution that gave a positive signal (>3-fold signal of the prebleeds). Animals that received the FCA/FIA (groups 1 and 2) or combination of Carbopol-971P and MF59 have significantly higher endpoint IgG titers than others (p<0.05).

Serum antigen-specific IgG was measured by ELISA at the terminal bleed, 4 weeks after the 3^rd^ immunization (Figure 3). Rabbits immunized without adjuvant (group 12) were used as our low-end benchmark. These animals developed a relatively high mean titer (6.4×10^5^) after three immunizations over the course of 16 weeks, despite no adjuvant being used. FCA/FIA adjuvants were used as the high-end benchmark, and as expected, groups 1 and 2 had the highest binding IgG titers among all groups (mean = 6.2–6.5×10^6^, p<0.05). Although animals in group 1 received 3-fold more gp140_SF162_ immunogen than those in group 2, there was no significant difference in the mean binding titers, suggesting that increasing the quantity of antigen does not offer additional benefits in the presence of a potent adjuvant. Among all other adjuvanted groups, only those that were immunized with the combination of Carbopol-971P and MF59 (group 10) elicited titers comparable to the FCA/FIA groups (mean = 5.8×10^6^, p = 1.00). When used individually, the titers elicited by MF59 or Carbopol-971P were 7- to 8-fold lower than the combination group or the FCA/FIA groups (p<0.001). Sequential immunization of Carbopol-971P followed by MF59 (group 6) or in reverse order (group 8) also resulted in titers similar to those of the single adjuvant protocols, indicating that concomitant use of both adjuvants was needed to enhance antibody titers. In addition, the high antibody titer induced by the combination group was very specific to the Carbopol-971P formulation, as the mean titer of those immunized with combination of Carbopol-974P and MF59 (group 11) was 6-fold lower in comparison (mean = 9.1×10^5^, p<0.005). Individually, Carbopol-971P appeared to be slightly more active and elicited higher binding titers of antibodies than Carbopol-974P (means = 7.24×10^5^ and 6.45×10^5^, respectively), but overall differences were not significant.

### Adjuvant increased antibody avidity

**Figure 4:**
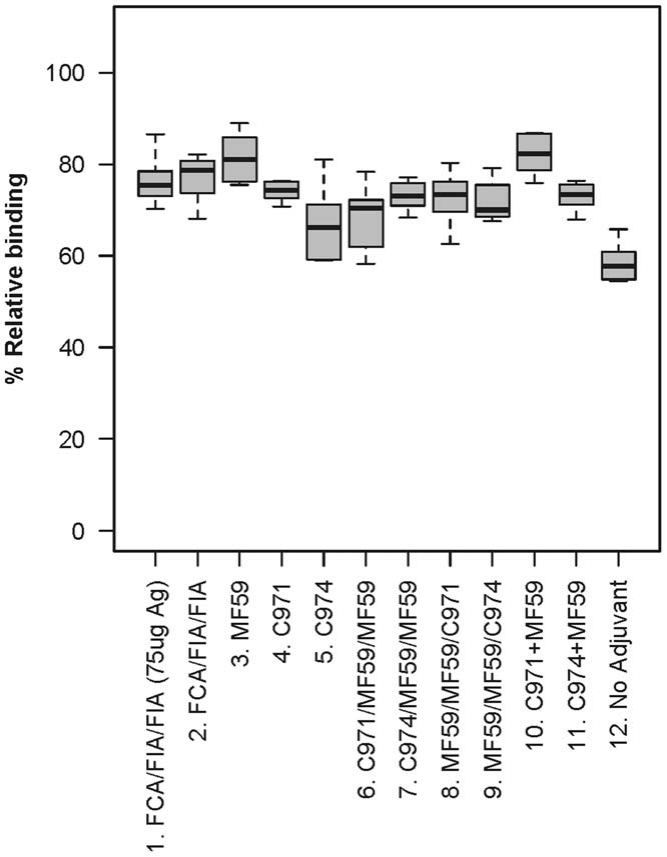
Relative avidity index. Avidity of the antiserum was measured by its ability to remain in binding with gp140_SF162_ in the presence of 8 M Urea. All samples were found to have relative high avidity, defined as >50% IgG binding in the presence of 8 M Urea, as compared to the no Urea control. Compared to the unadjuvanted group (no. 12), antisera from animals vaccinated with adjuvants have significantly higher RAI (p<0.05).

To estimate antibody affinity maturation, avidity of the antisera was measured by the 8 M urea displacement method as described previously [49]. Although all antisera tested have high relative avidity indices (RAI), defined as >50% residual binding, the mean RAI was significantly lower in those immunized without adjuvant (58.5%, p<0.05; Figure 4). The oil-in-water emulsion adjuvants, such as FCA/FIA and MF59, elicited antibodies with higher RAI, ranging from 71–82%, whether they were used alone or in combination with Carbopols. Comparing the two carbomers, antisera from rabbits immunized with Carbopol-971P had modestly higher avidity than those immunized with Carbopol-974P (mean = 74.0% and 66.7%, respectively; Figure 4), although the difference is not significant between the two groups. Again, as observed with IgG binding titer, increasing the immunogen dosage did not have any effect on the avidity, as the polyclonal antisera from groups 1 and 2 were found to have no significant differences in their RAI (mean = 76.5% and 76.9%, respectively; Figure 4).

### Epitope specificity of the antibodies varied depending on the adjuvants used

To investigate if antibodies elicited by different adjuvants were qualitatively distinct in the epitopes that they targeted, the polyclonal antisera were tested for their ability to compete with a panel of well-characterized mAbs in binding to gp140_SF162_. Two mAbs targeting each distinct antigenic site were used; these included b12 and VRC01 (CD4bs), 3019 and HGN194 (V3 loop), 17b and E51 (CD4i) and 2F5 and 4E10 (MPER). Antisera were normalized for endpoint IgG titers, such that similar amounts of polyclonal antibodies were used to compete each mAb. The comprehensive results are shown in Figure S2.

**Figure 5:**
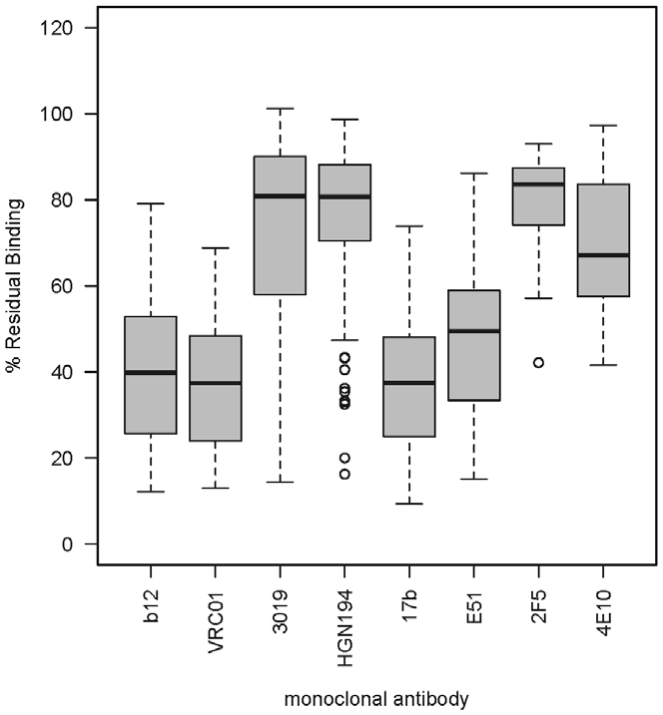
Epitope specificity across all antisera by mAb competition. Across all samples, the polyclonal antibody responses appeared be mostly CD4bs and CD4i driven, as the antisera could efficiently out-competed mAbs against these two regions (b12 and VRC01 for CD4bs and 17b and E51 for CD4i). Modest to little activities against the V3 loop or the MPER were detected.

**Figure 6:**
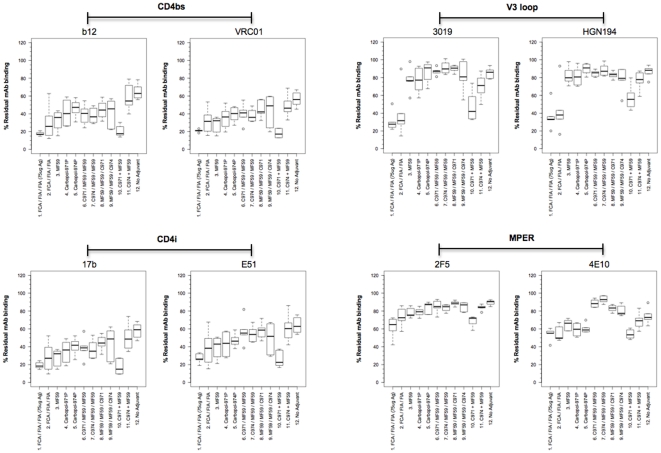
Antisera competition to different mAbs. Antiserum was used to compete with Eu-labeled mAbs specific for different antigenic domains of Env. The fluorescent signal of Europium is proportional to residual mAb binding, relative to the prebleed controls. A decrease in residual mAb binding indicated an increase in specificity of the antiserum for the epitope site. Both the FCA/FIA (groups 1&2) and the combination of Carbopol-971P and MF59 (group 10) displayed potent activities towards both CD4bs and CD4i and modest activities towards the V3 loop and MPER. By contrast, antisera from animals vaccinated with other adjuvants displayed only modest activities to CD4bs and CD4i and little, if any, towards V3 and MPER.

Across all groups, it appeared that most of the antibodies elicited were directed against the CD4bs and the CD4i regions of Env, as the antisera could out-compete up to 50% of the respective mAbs (Figure 5). Antisera from animals immunized with the combination of Carbopol-971P and MF59 (group 10) displayed the strongest activities to both CD4bs and CD4i, as they could potently out-compete an average of 80% of the four mAbs tested (shown as 20% residual mAb binding; Figure 6). To our surprise, binding activity to the V3 loop was only weakly detectable in most of the antisera, except those immunized with FCA/FIA (groups 1 and 2) or the combination of Carbopol-971P and MF59 (group 10), where the polyclonal antibodies modestly to potently out-competed both 3019 and HGN194 mAbs (Figure 6). Similarly, serum antibody binding to the MPER was either completely absent or only weakly detected. While animals immunized with FCA/FIA or combination of Carbopol-971P and MF59 (groups 1&2 and 10, respectively) competed with 2F5 and 4E10 binding to a modest degree, antisera from other groups, at best, only weakly out-competed 4E10 and showed limited, if any, competition to 2F5 (Figure 6).

### Neutralizing antibody responses

**Figure 7:**
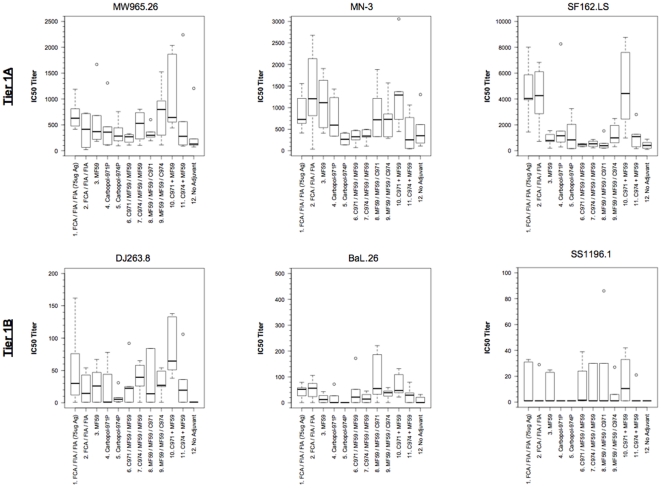
50% Neutralization titer of individual pseudovirus between groups. For single adjuvant, animals vaccinated with FCA/FIA (groups 1 and 2) have the highest titers against the tier 1A viruses, and low titers against tier 1B. Sequential immunization with different prime-boost combination of Carbopol-971P/974P and MF59 (groups 6–9) performed no better than using FCA/FIA or MF59 alone. By contrast, the combination of Carbopol-971P and MF59 (group 10) conferred significantly higher neutralization titers over all others (p<0.05), particularly against the tier 1B viruses DJ263.8 and SS1196.1.

To examine whether different adjuvant formulations had any effect on neutralization breadth and depth, rabbit antisera were assessed against a panel of tier 1 pseudoviruses using the standard TZM-bl assay (comprehensive results shown in Figure S3). Among animals immunized with either a single adjuvant or different adjuvants in sequential order, the neutralization titers against the autologous SF162.LS virus were the highest in animals immunized with FCA/FIA (groups 1 and 2) with mean group titers of 4,663 to 4,178, respectively (Figure 7). Although the neutralization titer was slightly higher in group 1 than group 2, the former of which received 3 times more antigen, the difference in titer was not significant. When formulated together, this mixture of Carbopol-971P and MF59 (group 10) was found to be highly immune stimulating, and elicited neutralizing titers higher than the FCA/FIA groups against SF162.LS (mean = 4,776; Figure 7). By contrast, the titers were at least 2-fold lower when MF59 (group 3) or Carbopol-971P (group 4) was used alone (mean = 885 and 2,175, respectively, p<0.05; Figure 7). The same effect was observed when the antisera were assayed with other pseudoviruses, in which the combination of Carbopol-971P and MF59 elicited the highest neutralization titers across all groups and against all pseudoviruses but BaL.26 (Figure 7). On the other hand, sequential immunization of Carbopol-971P followed by MF59 (group 6), or vice versa (group 8), did not have any additive or synergistic effect and the overall neutralization titers were either similar or lower than using either Carbopol-971P or MF59 alone (p>0.5; Figure 7). The increase in titers was specific for the 971P formulation of Carbopol, as combining Carbopol-974P and MF59 (group 11) resulted in significantly lower titers against all viruses tested (p<0.05). This observation might in part be due to the fact that Carbopol-974P (group 5) was less immunogenic than Carbopol-971P and elicited lower nAb titers on its own (Figure 7). Of note, even in the absence of adjuvant (group 17) the antigen was capable of eliciting low titer of nAbs against the readily neutralized tier 1A pseudoviruses, but the response was almost completely absent against the more neutralization resistant tier 1B viruses (Figure 7 and Figure S3).

## Discussion

An efficacious preventive vaccine should be one capable of inducing protective and systemic T-helper responses [51]. For HIV-1, the Env is the only antigenic determinant for nAb responses. However, recombinant gp140, peptide mimetics, structural scaffolds and other rationally designed immunogens have, thus far, failed to elicit antibodies that are broadly neutralizing with high titer and prolonged duration that have been observed in some HIV-1 patients [52]. To compensate for the poor immunogenicity of these subunit antigens, adjuvant has often been used to supplement immunogens. Although the precise mechanism of many adjuvants remains poorly understood, adjuvants generally act in one or more of the following ways to enhance immune responses [53], [54], [55]: (1) they function as a depot to prolong antigen release and presentation; (2) they act as a delivery vehicle to promote antigen uptake and presentation by antigen-presenting cells (APCs) and enhance subsequent trafficking to the lymph nodes; and (3) they activate innate immune signaling pathways (e.g. TLRs) and/or stimulate the release of pro-inflammatory cytokines.

Here, we have compared a panel of different adjuvants including MF59, Carbopol-971P and 974P, against FCA/FIA, for their ability to enhance humoral responses. Oil-in-water emulsions, such as FCA/FIA or other modified formulations, are known for potent adjuvant activities because they possess both depot forming and immunostimulatory properties [53], [55]. Indeed, animals that were immunized with gp140 in FCA/FIA adjuvant elicited high titers of nAb, particularly against the autologous SF162.LS virus (Figure 7 and Figure S3). The potent FCA/FIA adjuvant also displayed antigen-sparing properties, in which it was unnecessary to increase the antigen dosage in achieving the same degree of immune response (group 1 *vs.* group 2; Figure 7 and Figure S3). Despite possessing superior immunogenicity, oil-in-water emulsions can also be highly toxic and cause severe local and systemic adverse effects to vaccinees and cannot easily be used in prophylactic vaccines [20], [21], [22], [23]. The only emulsion adjuvant that has been approved for human use is MF59. While MF59 showed superior humoral responses over the classical alum adjuvants in a previous study with HBV antigen, it only modestly increased the antibody titers in the elderly, but not young adults, in the context of influenza vaccine [56]. In our hands, MF59 promoted a 2-fold increase in nAb titers, compared to unadjuvanted immunization with gp140_SF162_ (Figure 7 and Figure S3). However, nAb responses elicited by MF59 were up to 4-fold lower than using FCA/FIA, suggesting that reduction in reactogenicity also compromised adjuvanticity.

Due to safety concerns and existing body of pre-clinical and clinical data supporting MF59's use in its present composition [57], increasing the amount of active ingredient (i.e. Squalene) in MF59 may not a feasible option for enhancing its adjuvanticity. Since MF59 is an immunopotentiating adjuvant that elicits a Th2-type immune bias [27], [58], our strategy was to increase the dimension of adjuvant activity by including another adjuvant with both depot-forming ability and potent Th1-immune activating properties. Polymers such as Carbopols have long been used as controlled release agents in pharmaceuticals [38], [39], and more recently, as adjuvants in veterinary vaccines [40], [41], [42], [43], [59]. Among the two polymers in this study, Carbopol-971P contains fewer crosslinking sites and forms a flexible “fishnet” structure with little interstitial space between particles; as a result, it has a slower substance release mechanism [46], [47]. By contrast, Carbopol-974P is highly crosslinked into a “fuzzball” structure and is less efficient in controlling substance release [46], [47]. This difference in their structural flexibility and controlled release ability might affect the extent of the depot formation at the injection site, and thereby accounted for the different adjuvant activity observed. Individually, Carbopol-971P appeared to be more stimulatory than its 974P counterpart and elicited higher nAb titers (Figure 7 and Figure S3). When combined with MF59, Carbopol-974P offered little advantage over using MF59 alone. However, a combination of Carbopol-971P and MF59 showed superior potency over all other adjuvants, including FCA/FIA and had the highest nAb titers against 5/6 viruses tested (Figure 7 and Figure S3).

Since the immune pathway mediated by MF59 has not been fully characterized and that of Carbopols was poorly understood, substantial investigations are required to understand the mechanism behind the potent adjuvanticity of the Carbopol-971P and MF59 combination. Nevertheless, we speculate that the effect was due to simultaneous and synergic immunostimulation, as MF59 and Carbopol can each account for different types of immune response. In general, MF59 stimulates both Th1 and Th2 responses. It was shown to enhance differentiation of monocytes into DCs [29], promote local recruitment of monocytes, granulocytes and DCs [30], increase antigen uptake [28], [29] and facilitate antigen transport to the draining lymph nodes [30]. On the other hand, Carbopols have been used as antigen delivery vehicles and is associated to B-cell activation [60]. The anionic polymer also been shown to induce high titers of both Th1 and Th2 cytokines and mediate a Th1 isotype-switched antibody response [44]. We postulate that the difference in adjuvanticity between Carbopol-971P and 974P was due to greater structural flexibility and the slow release mechanism of the former, which can better prolong gp140_SF162_ antigen exposure to APCs, but also extend the duration in which MF59 stimulates and recruites local APCs for antigen uptake, thereby synergistically enhancing the humoral responses.

While the combination of Carbopol-971P and MF59 could induce significant increase in antibody response, sequential immunization with Carbopols followed by MF59 or vice versa failed to improve nAb titers over using MF59 or Carbopols alone (groups 6–9; Figure 7 and Figure S3). This observation leads to the speculation that if one or all of the adjuvants used in sequential immunization is stimulatory on its own with a distinct mechanism, then a prime-boost protocol may not provide enough stimuli to sufficiently mediate each response for a synergic effect, particularly if the immunizations were administered over a short time course like the one here.

Although the use of adjuvants, particularly those with limited immunogenicity, does not necessarily increase the overall antigen-specific IgG titers (Figure 3), they might at least facilitate the generation of high avidity antibodies (Figure 4), which are suggested to correlate with protection *in vivo* [1], [2], [3]. In addition, the more potent adjuvants, such as FCA/FIA, MF59 and the combination Carbopol-971P and MF59 enhanced nAb titers and magnified the responses to the V3 loops and MPER that were only weakly detectable with other adjuvants (Figure 6 and Figure S2). Finally, the significant increase in nAb titers elicited by the combination of Carbopol-971P and MF59 is universal, as subsequent immunization studies with different antigens in different species also raised superior nAb titers over other adjuvant protocols (unpublished data and [45]). Taken together, future studies will be undertaken with the Carbopol-971P and MF59 combination formulated with modified HIV-1 Env antigens, in effort to elicit nAbs targeting the most desired nAb epitopes.

## Supporting Information

Table S1 **Sample adjuvant formulation.** An example on formulation with different adjuvant(s) is detailed here. In this study, the gp140_SF162_ glycoprotein was concentrated to 1 µg/µl, such that 25 µg of antigen was equivalent to 25 µl. The volume of adjuvants used should be adjusted according to the volume of the antigen, depending on its concentration, in other cases. The final concentration and volume of each adjuvant, as well as the antigen-adjuvant mixture, was kept identical in all groups to allow direct comparison.
(TIF)

Figure S1 **Immunization protocol.** Twelve groups of 6 rabbits each were immunized with HIV-1 gp140_SF162_ at week 0, 4 and 12 with the adjuvants listed. Animals in group 1 received 75 µg of Env glycoprotein per injection, while all others received 25 µg of the same antigen. An unadjuvanted control group (no. 12) was included for baseline measure.
(TIF)

Figure S2 **Summary of mAb competition with antisera.** Summary of antisera competition with different mAbs is shown. Each treatment group consists of 6 rabbits (Rb), except for group 1 in which Rb5 died before the completion of the study. Results are color coded: Black represents less than 15% of residual mAb binding (or >85% displacement of mAb by the antiserum, relative to the prebleed); red is 16–30% residual mAb binding; orange is 31–50% and yellow is 51–75%. If the antiserum failed to out-compete at least 25% of the mAb (>75% residual mAb binding), they are considered negative and the value is uncolored.
(TIF)

Figure S3 **Neutralization titer by TZM-bl assay.** The 50% neutralization titer against a panel of tier 1A and 1B pseudovirus was determined by the TZM-bl assay. Each treatment group consists of 6 rabbits (Rb), except for group 1 in which Rb5 died before the completion of the study. The IC_50_ titer was considered true positive (colored) if it is at least 3-fold higher than the corresponding pre-bleeds (week −2) sample.
(TIF)

## References

[1] Devash Y, Calvelli TA, Wood DG, Reagan KJ, Rubinstein A (1990). Vertical transmission of human immunodeficiency virus is correlated with the absence of high-affinity/avidity maternal antibodies to the gp120 principal neutralizing domain.. Proc Natl Acad Sci U S A.

[2] Binley JM, Arshad H, Fouts TR, Moore JP (1997). An investigation of the high-avidity antibody response to glycoprotein 120 of human immunodeficiency virus type 1.. AIDS Res Hum Retroviruses.

[3] Zhao J, Lai L, Amara RR, Montefiori DC, Villinger F (2009). Preclinical studies of human immunodeficiency virus/AIDS vaccines: inverse correlation between avidity of anti-Env antibodies and peak postchallenge viremia.. J Virol.

[4] Mouquet H, Scheid JF, Zoller MJ, Krogsgaard M, Ott RG (2010). Polyreactivity increases the apparent affinity of anti-HIV antibodies by heteroligation.. Nature.

[5] Walker LM, Phogat SK, Chan-Hui PY, Wagner D, Phung P (2009). Broad and potent neutralizing antibodies from an African donor reveal a new HIV-1 vaccine target.. Science.

[6] Huber M, Le KM, Doores KJ, Fulton Z, Stanfield RL (2010). Very few substitutions in a germ line antibody are required to initiate significant domain exchange.. J Virol.

[7] Xiao X, Chen W, Feng Y, Zhu Z, Prabakaran P (2009). Germline-like predecessors of broadly neutralizing antibodies lack measurable binding to HIV-1 envelope glycoproteins: implications for evasion of immune responses and design of vaccine immunogens.. Biochem Biophys Res Commun.

[8] Corti D, Langedijk JP, Hinz A, Seaman MS, Vanzetta F (2010). Analysis of memory B cell responses and isolation of novel monoclonal antibodies with neutralizing breadth from HIV-1-infected individuals.. PLoS One.

[9] Wu X, Yang ZY, Li Y, Hogerkorp CM, Schief WR (2010). Rational design of envelope identifies broadly neutralizing human monoclonal antibodies to HIV-1.. Science.

[10] Wu X, Zhou T, Zhu J, Zhang B, Georgiev I (2011). Focused evolution of HIV-1 neutralizing antibodies revealed by structures and deep sequencing.. Science.

[11] Halperin SA, Dobson S, McNeil S, Langley JM, Smith B (2006). Comparison of the safety and immunogenicity of hepatitis B virus surface antigen co-administered with an immunostimulatory phosphorothioate oligonucleotide and a licensed hepatitis B vaccine in healthy young adults.. Vaccine.

[12] Boyle J, Eastman D, Millar C, Camuglia S, Cox J (2007). The utility of ISCOMATRIX adjuvant for dose reduction of antigen for vaccines requiring antibody responses.. Vaccine.

[13] Huleatt JW, Jacobs AR, Tang J, Desai P, Kopp EB (2007). Vaccination with recombinant fusion proteins incorporating Toll-like receptor ligands induces rapid cellular and humoral immunity.. Vaccine.

[14] Banzhoff A, Gasparini R, Laghi-Pasini F, Staniscia T, Durando P (2009). MF59-adjuvanted H5N1 vaccine induces immunologic memory and heterotypic antibody responses in non-elderly and elderly adults.. PLoS One.

[15] Galli G, Hancock K, Hoschler K, DeVos J, Praus M (2009). Fast rise of broadly cross-reactive antibodies after boosting long-lived human memory B cells primed by an MF59 adjuvanted prepandemic vaccine.. Proc Natl Acad Sci U S A.

[16] Schwarz TF, Horacek T, Knuf M, Damman HG, Roman F (2009). Single dose vaccination with AS03-adjuvanted H5N1 vaccines in a randomized trial induces strong and broad immune responsiveness to booster vaccination in adults.. Vaccine.

[17] O'Hagan DT (2000). Vaccine adjuvants: preparation methods and research protocols.

[18] Miller LH, Saul A, Mahanty S (2005). Revisiting Freund's incomplete adjuvant for vaccines in the developing world.. Trends Parasitol.

[19] Aucouturier J, Dupuis L, Deville S, Ascarateil S, Ganne V (2002). Montanide ISA 720 and 51: a new generation of water in oil emulsions as adjuvants for human vaccines.. Expert Rev Vaccines.

[20] Wu Y, Ellis RD, Shaffer D, Fontes E, Malkin EM (2008). Phase 1 trial of malaria transmission blocking vaccine candidates Pfs25 and Pvs25 formulated with montanide ISA 51.. PLoS One.

[21] Genton B, Al-Yaman F, Anders R, Saul A, Brown G (2000). Safety and immunogenicity of a three-component blood-stage malaria vaccine in adults living in an endemic area of Papua New Guinea.. Vaccine.

[22] Toledo H, Baly A, Castro O, Resik S, Laferte J (2001). A phase I clinical trial of a multi-epitope polypeptide TAB9 combined with Montanide ISA 720 adjuvant in non-HIV-1 infected human volunteers.. Vaccine.

[23] Graham BS, McElrath MJ, Keefer MC, Rybczyk K, Berger D (2010). Immunization with cocktail of HIV-derived peptides in montanide ISA-51 is immunogenic, but causes sterile abscesses and unacceptable reactogenicity.. PLoS One.

[24] Glenny AT (1930). Insoluble Precipitates in Diphtheria and Tetanus Immunization.. Br Med J.

[25] Gregoriadis G, Allison AC, Poste G, North Atlantic Treaty Organization. Scientific Affairs Division (1989). Immunological adjuvants and vaccines.

[26] Podda A, Del Giudice G (2003). MF59-adjuvanted vaccines: increased immunogenicity with an optimal safety profile.. Expert Rev Vaccines.

[27] Ott G, Barchfeld GL, Chernoff D, Radhakrishnan R, van Hoogevest P (1995). MF59. Design and evaluation of a safe and potent adjuvant for human vaccines.. Pharm Biotechnol.

[28] Dupuis M, Murphy TJ, Higgins D, Ugozzoli M, van Nest G (1998). Dendritic cells internalize vaccine adjuvant after intramuscular injection.. Cell Immunol.

[29] Seubert A, Monaci E, Pizza M, O'Hagan DT, Wack A (2008). The adjuvants aluminum hydroxide and MF59 induce monocyte and granulocyte chemoattractants and enhance monocyte differentiation toward dendritic cells.. J Immunol.

[30] Calabro S, Tortoli M, Baudner BC, Pacitto A, Cortese M (2011). Vaccine adjuvants alum and MF59 induce rapid recruitment of neutrophils and monocytes that participate in antigen transport to draining lymph nodes.. Vaccine.

[31] Traquina P, Morandi M, Contorni M, Van Nest G (1996). MF59 adjuvant enhances the antibody response to recombinant hepatitis B surface antigen vaccine in primates.. J Infect Dis.

[32] Heineman TC, Clements-Mann ML, Poland GA, Jacobson RM, Izu AE (1999). A randomized, controlled study in adults of the immunogenicity of a novel hepatitis B vaccine containing MF59 adjuvant.. Vaccine.

[33] Pass RF, Duliege AM, Boppana S, Sekulovich R, Percell S (1999). A subunit cytomegalovirus vaccine based on recombinant envelope glycoprotein B and a new adjuvant.. J Infect Dis.

[34] Langenberg AG, Burke RL, Adair SF, Sekulovich R, Tigges M (1995). A recombinant glycoprotein vaccine for herpes simplex virus type 2: safety and immunogenicity [corrected].. Ann Intern Med.

[35] Kahn JO, Sinangil F, Baenziger J, Murcar N, Wynne D (1994). Clinical and immunologic responses to human immunodeficiency virus (HIV) type 1SF2 gp120 subunit vaccine combined with MF59 adjuvant with or without muramyl tripeptide dipalmitoyl phosphatidylethanolamine in non-HIV-infected human volunteers.. J Infect Dis.

[36] Nitayaphan S, Khamboonruang C, Sirisophana N, Morgan P, Chiu J (2000). A phase I/II trial of HIV SF2 gp120/MF59 vaccine in seronegative thais.AFRIMS-RIHES Vaccine Evaluation Group. Armed Forces Research Institute of Medical Sciences and the Research Institute for Health Sciences.. Vaccine.

[37] Cunningham CK, Wara DW, Kang M, Fenton T, Hawkins E (2001). Safety of 2 recombinant human immunodeficiency virus type 1 (HIV-1) envelope vaccines in neonates born to HIV-1-infected women.. Clin Infect Dis.

[38] Wade A, Weller PJ (1994). Handbook of pharmaceutical excipients.

[39] Blanco-Fuente H, Anguiano-Igea S, Otero-Espinar FJ, Blanco-Mendez J (1996). In-vitro bioadhesion of carbopol hydrogels.. Int J Pharm.

[40] Gualandi GL, Losio NM, Muratori G, Foni E (1988). The ability by different preparations of porcine parvovirus to enhance humoral immunity in swine and guinea pigs.. Microbiologica.

[41] Mumford JA, Wilson H, Hannant D, Jessett DM (1994). Antigenicity and immunogenicity of equine influenza vaccines containing a Carbomer adjuvant.. Epidemiol Infect.

[42] Elicker S, Sipos W (2009). The tissue compatibility of different Mycoplasma hyopneumoniae vaccines is mainly dependent upon their adjuvants.. Berl Munch Tierarztl Wochenschr.

[43] (2010). Suvaxyn M.hyo Suspension for injection for pigs.. http://wwwnoahcompendiumcouk/Pfizer_Limited/documents/S3979html.

[44] Krashias G, Simon AK, Wegmann F, Kok WL, Ho LP (2010). Potent adaptive immune responses induced against HIV-1 gp140 and influenza virus HA by a polyanionic carbomer.. Vaccine.

[45] Dey AK, Burke B, Sun Y, Hartog K, Heeney JL (2012). Use of a polyanionic carbomer, Carbopol971P, in combination with MF59, improves antibody responses to HIV-1 envelope glycoprotein.. Vaccine.

[46] Karsa DRE, Stephenson RAE (1996).

[47] Bonacucina G, Martelli S, Palmieri GF (2004). Rheological, mucoadhesive and release properties of Carbopol gels in hydrophilic cosolvents.. Int J Pharm.

[48] Srivastava IK, Stamatatos L, Legg H, Kan E, Fong A (2002). Purification and characterization of oligomeric envelope glycoprotein from a primary R5 subtype B human immunodeficiency virus.. J Virol.

[49] Cole KS, Rowles JL, Jagerski BA, Murphey-Corb M, Unangst T (1997). Evolution of envelope-specific antibody responses in monkeys experimentally infected or immunized with simian immunodeficiency virus and its association with the development of protective immunity.. J Virol.

[50] Li M, Gao F, Mascola JR, Stamatatos L, Polonis VR (2005). Human immunodeficiency virus type 1 env clones from acute and early subtype B infections for standardized assessments of vaccine-elicited neutralizing antibodies.. J Virol.

[51] Heeney JL (2004). Requirement of diverse T-helper responses elicited by HIV vaccines: induction of highly targeted humoral and CTL responses.. Expert Rev Vaccines.

[52] Mascola JR, Montefiori DC (2010). The role of antibodies in HIV vaccines.. Annu Rev Immunol.

[53] Gupta RK, Siber GR (1995). Adjuvants for human vaccines–current status, problems and future prospects.. Vaccine.

[54] Coffman RL, Sher A, Seder RA (2010). Vaccine adjuvants: putting innate immunity to work.. Immunity.

[55] Manmohan Singh N (2007). Vaccine adjuvants and delivery systems.

[56] Mbow ML, De Gregorio E, Valiante NM, Rappuoli R (2010). New adjuvants for human vaccines.. Curr Opin Immunol.

[57] O'Hagan DT (2007). MF59 is a safe and potent vaccine adjuvant that enhances protection against influenza virus infection.. Expert Rev Vaccines.

[58] Dey AK, Srivastava IK (2011). Novel adjuvants and delivery systems for enhancing immune responses induced by immunogens.. Expert Rev Vaccines.

[59] Opriessnig T, Fenaux M, Thomas P, Hoogland MJ, Rothschild MF (2006). Evidence of breed-dependent differences in susceptibility to porcine circovirus type-2-associated disease and lesions.. Vet Pathol.

[60] Cranage MP, Fraser CA, Cope A, McKay PF, Seaman MS (2011). Antibody responses after intravaginal immunisation with trimeric HIV-1 CN54 clade C gp140 in Carbopol gel are augmented by systemic priming or boosting with an adjuvanted formulation.. Vaccine.

