# Supplementary figures and images for: Mixed Adjuvant Formulations Reveal a New Combination That Elicit Antibody Response Comparable to Freund's Adjuvants
Source: PLoS One. 2012 Apr 11;7(4):e35083. doi: 10.1371/journal.pone.0035083 (PMC3324409; doi:10.1371/journal.pone.0035083)

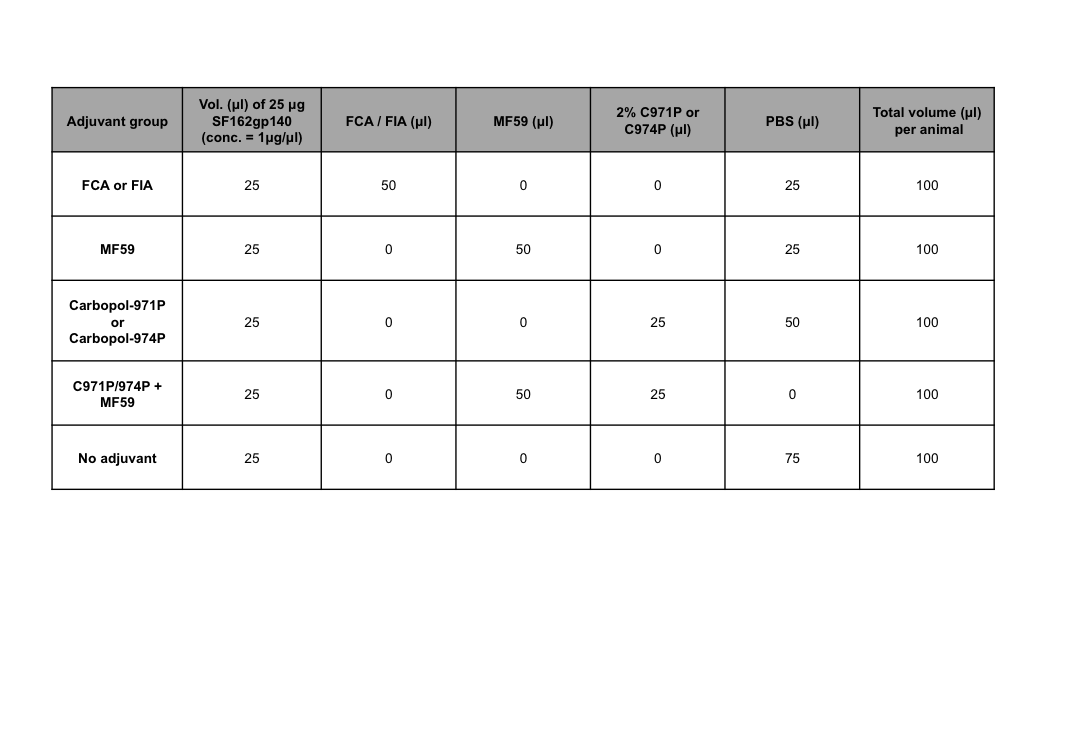

Supplement: Table S1 — Sample adjuvant formulation. An example on formulation with different adjuvant(s) is detailed here. In this study, the gp140SF162 glycoprotein was concentrated to 1 µg/µl, such that 25 µg of antigen was equivalent to 25 µl. The volume of adjuvants used should be adjusted according to the volume of the antigen, depending on its concentration, in other cases. The final concentration and volume of each adjuvant, as well as the antigen-adjuvant mixture, was kept identical in all groups to allow direct comparison. (TIF) [file pone.0035083.s001.tif]

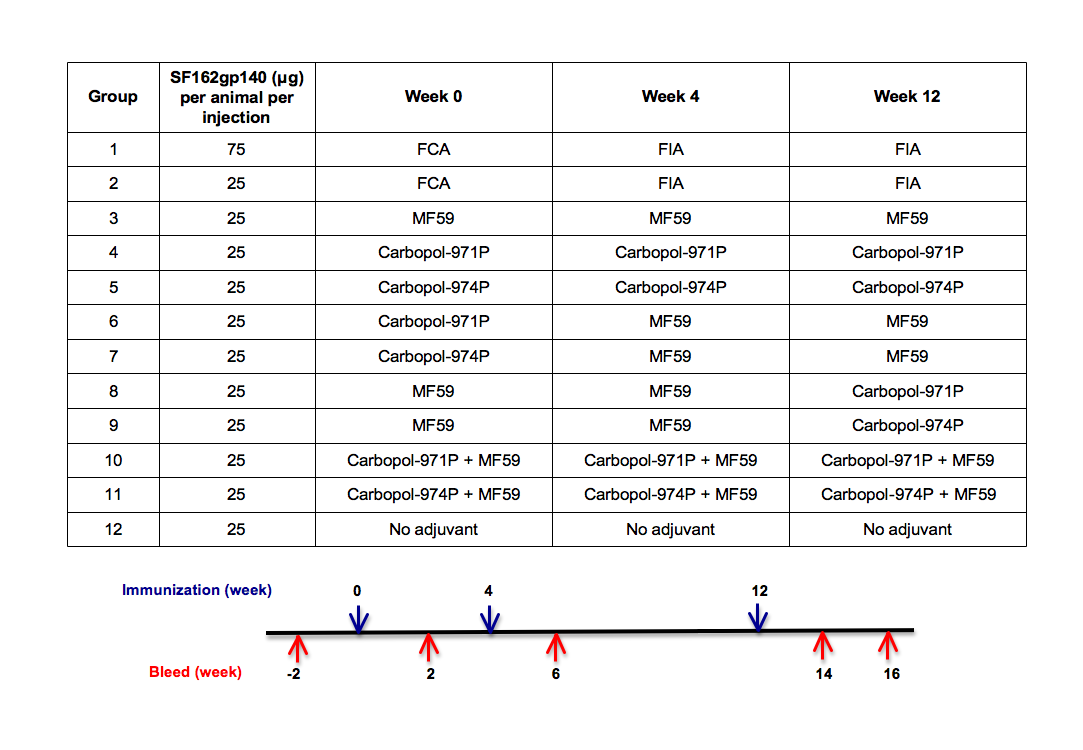

Supplement: Figure S1 — Immunization protocol. Twelve groups of 6 rabbits each were immunized with HIV-1 gp140SF162 at week 0, 4 and 12 with the adjuvants listed. Animals in group 1 received 75 µg of Env glycoprotein per injection, while all others received 25 µg of the same antigen. An unadjuvanted control group (no. 12) was included for baseline measure. (TIF) [file pone.0035083.s002.tif]

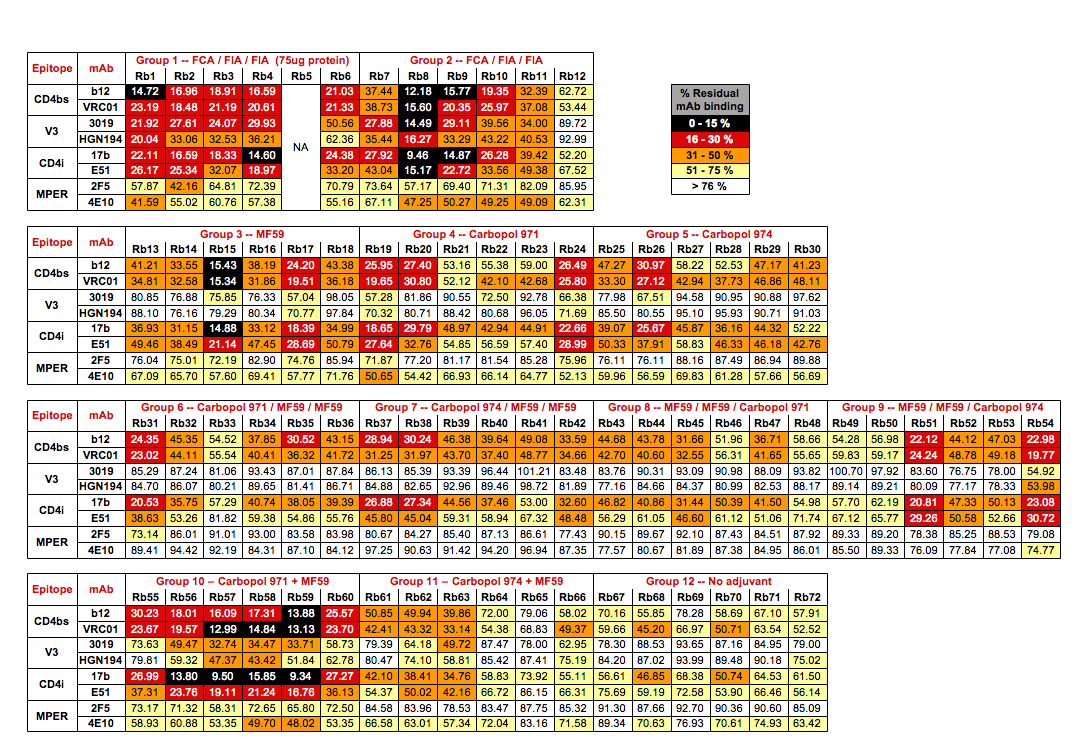

Supplement: Figure S2 — Summary of mAb competition with antisera. Summary of antisera competition with different mAbs is shown. Each treatment group consists of 6 rabbits (Rb), except for group 1 in which Rb5 died before the completion of the study. Results are color coded: Black represents less than 15% of residual mAb binding (or >85% displacement of mAb by the antiserum, relative to the prebleed); red is 16–30% residual mAb binding; orange is 31–50% and yellow is 51–75%. If the antiserum failed to out-compete at least 25% of the mAb (>75% residual mAb binding), they are considered negative and the value is uncolored. (TIF) [file pone.0035083.s003.tif]

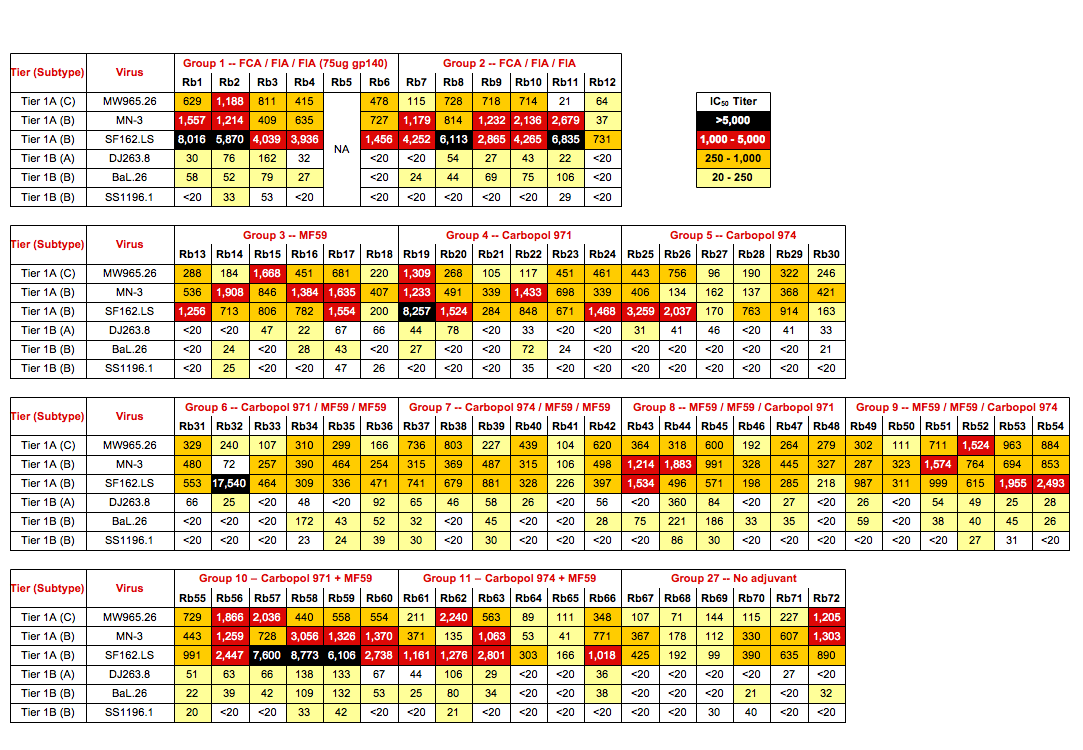

Supplement: Figure S3 — Neutralization titer by TZM-bl assay. The 50% neutralization titer against a panel of tier 1A and 1B pseudovirus was determined by the TZM-bl assay. Each treatment group consists of 6 rabbits (Rb), except for group 1 in which Rb5 died before the completion of the study. The IC50 titer was considered true positive (colored) if it is at least 3-fold higher than the corresponding pre-bleeds (week −2) sample. (TIF) [file pone.0035083.s004.tif]
